# Steady-State Analysis of a Flexible Markovian Queue with Server Breakdowns

**DOI:** 10.3390/e21030259

**Published:** 2019-03-07

**Authors:** Messaoud Bounkhel, Lotfi Tadj, Ramdane Hedjar

**Affiliations:** 1 Department of Mathematics, College of Science, King Saud University, P.O. Box 2455, Riyadh 11451, Saudi Arabia; 2 Department of Industrial Engineering, Alfaisal University, Riyadh 12714, Saudi Arabia; 3 Department of Computer Engineering, King Saud University, Riyadh 11453, Saudi Arabia

**Keywords:** Markovian queue, flexible server, unreliable server, steady-state distribution, linear operator

## Abstract

A flexible single-server queueing system is considered in this paper. The server adapts to the system size by using a strategy where the service provided can be either single or bulk depending on some threshold level *c*. If the number of customers in the system is less than *c*, then the server provides service to one customer at a time. If the number of customers in the system is greater than or equal to *c*, then the server provides service to a group of *c* customers. The service times are exponential and the service rates of single and bulk service are different. While providing service to either a single or a group of customers, the server may break down and goes through a repair phase. The breakdowns follow a Poisson distribution and the breakdown rates during single and bulk service are different. Also, repair times are exponential and repair rates during single and bulk service are different. The probability generating function and linear operator approaches are used to derive the system size steady-state probabilities.

## 1. Introduction

Markovian queueing models have Poisson arrivals and exponential service times. Because they are (arguably) easy to analyze, they are often used as a first step in analyzing more difficult queueing systems. They also yield practical results that present no difficulty in implementation. Early models were Markovian and found application in the telephone industry. However, since then, they also found other areas of application such as computer, transportation, and production systems.

The literature on Markovian queueing systems is huge. To cite a few of the latest, Wang et al. [1] use a Markovian queue to model a passenger-taxi system. They design a social benefit function and look for the system parameters that optimize the system operations.

Jain et al. [2] investigate a theoretical model of a Markovian system where the server takes vacations. During a vacation, the server does not stop serving customers but reduces his service rate. Also, customers can get discouraged and may not join the queue. Jain et al. study the transient behavior of the system using probability generating functions.

While researchers use Shannon entropy to measure randomness in queueing systems, Srivastava [3] uses Renyi’s measure of entropy to quantify uncertainty in Markovian queueing systems with finite and infinite capacity.

Estimation of the parameters of Markovian queueing systems using statistical techniques and simulation is also reported in numerous papers, see for example Refs. [4,5,6].

Some adaptive queueing systems have been considered in the literature. For example, Di Crescenzo et al. [7] provides an example of a queueing system working under two alternating regimes. Also, in many queueing systems, the server is prone to failure, for example Krishna Kumar et al. [8], Choudhury and Tadj [9], Kalidass et al. [10], Ammar [11], and Di Crescenzo et al. [12,13] deal with queueing models subject to failures (breakdowns) and repairs.

Usually, the interest in studying queueing systems is in obtaining the steady-state system size probabilities and, often, these are obtained in the form of a probability generating function (PGF). However, to recover the individual probability, one needs to calculate successive derivatives of the PGF. To avoid calculating such derivatives researchers resort to numerical methods. For example, Tadj and Hamdi [14] employ the maximum entropy approach to a quorum queueing system. Various numerical techniques were used by Lotfi Tadj and Chakib Tadj [15] to the same system. Also, Tadj [16,17] find the steady-state system size probabilities in terms of the zeros of a characteristic equation inside and outside the unit ball.

In this paper we consider a Markovian queueing system and utilize a numerical method using operators to obtain the steady-state system size probabilities and the analytical approach to obtain the PGF of these same probabilities. The system under study is quite versatile and is described in Section 2. Analysis of the two methods are carried out in Section 3. Section 4 describes a case study and the paper is concluded in Section 4.

## 2. Model Formulation

Oftentimes, a service or manufacturing firm can process its customers either singly or in batches, see case study below. It may not be economical to process customers singly, and it may be impossible to always process them in batches. In this case, a service discipline where the firm decides to process customers singly at times and in batches at other times is more appropriate. We therefore consider in this paper a single-server Markovian queueing system where the switch from one service discipline to the other is triggered by some constant integer c≥2. If the number of customers in this system is less than *c*, then the server processes customers one at a time (single mode). If the number of customers in greater than or equal to *c*, then the server provides service simultaneously to a group of *c* of customers (batch mode).

To define the rest of the notation, we assume that customer arrivals follow a Poisson process with positive rate λ. In single mode (i.e., when the number of customers in the system is less than *c*), service follows an exponential distribution with positive rate $\mu_{1}$. In batch mode (i.e., when the number of customers in the system is greater than *c*), service follows an exponential distribution with positive rate $\mu_{2}>\mu_{1}$. To mark the transition from single mode to batch mode, we assume that when the number of customers in the system is equal to *c*, then service is batch but with rate $\mu_{2}-\mu_{1}$. We also assume that the server is unreliable and may break down, either in single mode or in batch mode. Breakdowns occur according to a Poisson process with positive rate $\alpha_{1}$ in single mode and $\alpha_{2}$ in batch mode. A breakdown is followed by a repair of the server and repair times follow an exponential distribution with positive rate $\beta_{1}$ in single mode and $\beta_{2}$ in batch mode. When the server is unavailable, customers are allowed to join the queue in single mode, but not in batch mode, to avoid large queue lengths.

**Figure 1:**
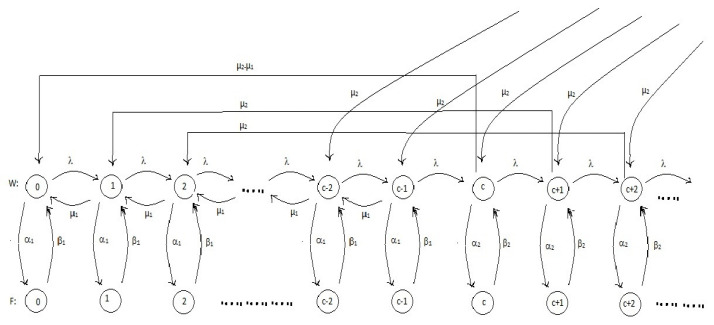
Rate transition diagram.

Let X(t) denote the number of customers in the system at time *t* and $P_{n}\left(t\right),n=0,1,2,\cdots$ denote the probability of *n* customers in the system at time *t*. The process {X(t);t≥0} is a continuous-time Markov chain, and the corresponding rate-transition diagram is depicted in Figure 1.

Since the server may be either working or down, we introduce $W_{n}\left(t\right),n=0,1,2,\cdots$ the probability of *n* customers in the system at time *t* when the server is in a working state, and $F_{n}\left(t\right),n=0,1,2,\cdots$ the probability of *n* customers in the system at time *t* when the server is in a failing state. Note that we readily have $P_{n}\left(t\right)=W_{n}\left(t\right)+F_{n}\left(t\right)$, the probability of *n* customers in the system at time *t*, regardless of the server state.

Writing the Chapman–Kolmogorov equations in the case where the server is in a working state, we have

(1) $$\frac{d}{dt}W_{0}\left(t\right)=-\left(\lambda +\alpha_{1}\right)W_{0}\left(t\right)+\beta_{1}F_{0}\left(t\right)+\mu_{1}W_{1}\left(t\right)+\left(\mu_{2}-\mu_{1}\right)W_{c}\left(t\right),$$

(2) $$\begin{array}{ccc} \frac{d}{dt}W_{n}\left(t\right) & = & -\left(\lambda +\alpha_{1}+\mu_{1}\right)W_{n}\left(t\right)+\lambda W_{n-1}\left(t\right)+\mu_{1}W_{n+1}\left(t\right)+\mu_{2}W_{n+c}\left(t\right),\\ & & +\beta_{1}F_{n}\left(t\right),\;\;\;\;\;\;\;\;\;\;\;\;\;\;1\leq n\leq c-1, \end{array}$$

(3) $$\frac{d}{dt}W_{n}\left(t\right)=-\left(\lambda +\alpha_{2}+\mu_{2}\right)W_{n}\left(t\right)+\lambda W_{n-1}\left(t\right)+\mu_{2}W_{n+c}\left(t\right)+\beta_{2}F_{n}\left(t\right),\;n\geq c.$$

Similarly, writing the Chapman–Kolmogorov equations in the case where the server is in a failing state, we have

(4) $$\frac{d}{dt}F_{0}\left(t\right)=-\beta_{1}F_{0}\left(t\right)+\alpha_{1}W_{0}\left(t\right),$$

(5) $$\frac{d}{dt}F_{n}\left(t\right)=-\beta_{1}F_{n}\left(t\right)+\alpha_{1}W_{n}\left(t\right),\;1\leq n\leq c-1,$$

(6) $$\frac{d}{dt}F_{n}\left(t\right)=-\beta_{2}F_{n}\left(t\right)+\alpha_{2}W_{n}\left(t\right),\;n\geq c.$$

Taking the limit in both sets of difference-differential equations as t→∞ yields the balance equations

(7) $$\left(\lambda +\alpha_{1}\right)W_{0}=\beta_{1}F_{0}+\mu_{1}W_{1}+\left(\mu_{2}-\mu_{1}\right)W_{c},$$

(8) $$\left(\lambda +\alpha_{1}+\mu_{1}\right)W_{n}=\lambda W_{n-1}+\mu_{1}W_{n+1}+\mu_{2}W_{n+c}+\beta_{1}F_{n},\;1\leq n\leq c-1,$$

(9) $$\left(\lambda +\alpha_{2}+\mu_{2}\right)W_{n}=\lambda W_{n-1}+\mu_{2}W_{n+c}+\beta_{2}F_{n},\;n\geq c,$$

(10) $$\beta_{1}F_{0}=\alpha_{1}W_{0},$$

(11) $$\beta_{1}F_{n}=\alpha_{1}W_{n},\;1\leq n\leq c-1,$$

(12) $$\beta_{2}F_{n}=\alpha_{2}W_{n},\;n\geq c.$$

By substitution of (10), (11), and (12) into (7), (8), and (9), respectively, we obtain

(13) $$\lambda W_{0}=\mu_{1}W_{1}+\left(\mu_{2}-\mu_{1}\right)W_{c},$$

(14) $$\left(\lambda +\mu_{1}\right)W_{n}=\lambda W_{n-1}+\mu_{1}W_{n+1}+\mu_{2}W_{n+c},\;1\leq n\leq c-1,$$

(15) $$\left(\lambda +\mu_{2}\right)W_{n}=\lambda W_{n-1}+\mu_{2}W_{n+c},\;n\geq c.$$

These difference equations are solved in the next section.

## 3. Model Solution

We use two methods to solve the set of Equations (13)–(15). The first method involves probability generating functions (PGF). The second one involves the concept of linear operators. Our aim is to solve the considered problem using the above two different methods and then to compare the obtained probabilities.

### 3.1. Analytical Method Using PGF

The procedure is to find a closed-form expression for the PGF. Then, if possible, expand it as a power series. If not, the probabilities are obtained through successive differentiations.

#### 3.1.1. Computations of the Probabilities $P_{n}$

In this method, to find the steady state probabilities $W_{n},\;F_{n},$ and $P_{n}$, we introduce for |z|≤1 the probability generating functions:

(16) $$W\left(z\right)=\sum_{n=0}^{\infty }W_{n}z^{n},\;F\left(z\right)=\sum_{n=0}^{\infty }F_{n}z^{n},\;P\left(z\right)=\sum_{n=0}^{\infty }P_{n}z^{n}.$$

To start, we define

$$S_{1}\left(z\right):=\sum_{n=0}^{c-1}W_{n}z^{n}\;\mathrm{and}\;S_{2}\left(z\right):=\sum_{n=c}^{2c-1}W_{n}z^{n}.$$

We multiply, for 1≤n≤c−1, both sides of Equation (14) by $z^{n+c}$:

(17) $$z^{c}\left(\lambda +\mu_{1}\right)W_{n}z^{n}=\lambda z^{c+1}W_{n-1}z^{n-1}+\mu_{1}z^{c-1}W_{n+1}z^{n+1}+\mu_{2}W_{n+c}z^{n+c}.$$

Taking the summation of these equations over *n* from 1 to c−1 yields:

(18) $$\begin{array}{ccc} z^{c}\left(\lambda +\mu_{1}\right)\sum_{n=1}^{c-1}W_{n}z^{n} & = & \lambda z^{c+1}\sum_{n=1}^{c-1}W_{n-1}z^{n-1}+\mu_{1}z^{c-1}\sum_{n=1}^{c-1}W_{n+1}z^{n+1}\\ & & +\mu_{2}\sum_{n=1}^{c-1}W_{n+c}z^{n+c}, \end{array}$$

which can be rewritten

(19) $$\begin{array}{ccc} z^{c}\left(\lambda +\mu_{1}\right)\left[S_{1}\left(z\right)-W_{0}\right] & = & \lambda z^{c+1}\left[S_{1}\left(z\right)-W_{c-1}z^{c-1}\right]+\mu_{2}\left[S_{2}\left(z\right)-W_{c}z^{c}\right]\\ & & +\mu_{1}z^{c-1}\left[S_{1}\left(z\right)-W_{0}-W_{1}z+W_{c}z^{c}\right]. \end{array}$$

Now, we multiply, for n≥c, both sides of Equation (15) by $z^{n+c}$:

(20) $$z^{c}\left(\lambda +\mu_{2}\right)W_{n}z^{n}=\lambda z^{c+1}W_{n-1}z^{n-1}+\mu_{2}W_{n+c}z^{n+c},\;n\geq c.$$

Similarly to the previous case, we take the summation of these equations over *n* from *c* to ∞ to get the following:

(21) $$z^{c}\left(\lambda +\mu_{2}\right)\sum_{n=c}^{\infty }W_{n}z^{n}=\lambda z^{c+1}\sum_{n=c}^{\infty }W_{n-1}z^{n-1}+\mu_{2}\sum_{n=c}^{\infty }W_{n+c}z^{n+c},$$

which is equivalent to

(22) $$\begin{array}{ccc} z^{c}\left(\lambda +\mu_{2}\right)\left[W\left(z\right)-S_{1}\left(z\right)\right] & = & \lambda z^{c+1}\left[W\left(z\right)-S_{1}\left(z\right)+W_{c-1}z^{c-1}\right]\\ & & +\mu_{2}\left[W\left(z\right)-S_{1}\left(z\right)-S_{2}\left(z\right)\right]. \end{array}$$

Rearranging terms, we have

(23) $$\begin{array}{ccc} \left[z^{c}\left(\lambda +\mu_{2}\right)-\lambda z^{c+1}-\mu_{2}\right]W\left(z\right) & = & \left[z^{c}\left(\lambda +\mu_{2}\right)-\lambda z^{c+1}-\mu_{2}\right]S_{1}\left(z\right)\\ & & +\lambda W_{c-1}z^{2c}-\mu_{2}S_{2}\left(z\right). \end{array}$$

From Equation (19) we have

(24) $$\begin{array}{ccc} \mu_{2}S_{2}\left(z\right) & = & \left[-\lambda z^{2}+\left(\lambda +\mu_{1}\right)z-\mu_{1}\right]z^{c-1}S_{1}\left(z\right)-\left[\left(\lambda +\mu_{1}\right)z-\mu_{1}\right]z^{c-1}W_{0}\\ & & +\mu_{1}z^{c}W_{1}+\lambda z^{2c}W_{c-1}+\left(\mu_{2}-\mu_{1}z^{c-1}\right)z^{c}W_{c}. \end{array}$$

Now replace $\mu_{2}S_{2}\left(z\right)$ with its expression in (23) to obtain:

$$\begin{array}{c} \left[z^{c}\left(\lambda +\mu_{2}\right)-\lambda z^{c+1}-\mu_{2}\right]W\left(z\right)=\left[z^{c}\left(\lambda +\mu_{2}\right)-\lambda z^{c+1}-\mu_{2}\right]S_{1}\left(z\right) \end{array}$$

$$\begin{array}{c} -\{\left[-\lambda z^{2}+\left(\lambda +\mu_{1}\right)z-\mu_{1}\right]z^{c-1}S_{1}\left(z\right)-\left[\left(\lambda +\mu_{1}\right)z-\mu_{1}\right]z^{c-1}W_{0}\\ +\mu_{1}z^{c}W_{1}+\lambda z^{2c}W_{c-1}+\left(\mu_{2}-\mu_{1}z^{c-1}\right)z^{c}W_{c}\}+\lambda W_{c-1}z^{2c}. \end{array}$$

Simplifying the RHS of this expression then solving for W(z), we get the PGF of the working steady-state probabilities

$$\begin{array}{c} W\left(z\right)=\frac{A_{1}\left(z\right)\sum_{n=0}^{n=c-1}W_{n}z^{n}+\left[\left(\lambda +\mu_{1}\right)z-\mu_{1}\right]W_{0}z^{c-1}-\mu_{1}z^{c}W_{1}+\left(\mu_{1}z^{c-1}-\mu_{2}\right)z^{c}W_{c}}{z^{c}\left(\lambda +\mu_{2}\right)-\lambda z^{c+1}-\mu_{2}}, \end{array}$$

where $A_{1}\left(z\right):=\left(\mu_{2}-\mu_{1}\right)z^{c}+\mu_{1}z^{c-1}-\mu_{2}$. Finally, using (7) we get the following

(25) $$\begin{array}{c} W\left(z\right)=\frac{A_{1}\left(z\right)\sum_{n=0}^{n=c-1}W_{n}z^{n}+A_{2}\left(z\right)W_{0}z^{c-1}+A_{3}\left(z\right)z^{c}W_{1}}{z^{c}\left(\lambda +\mu_{2}\right)-\lambda z^{c+1}-\mu_{2}}, \end{array}$$

where $A_{2}\left(z\right):=\left(\lambda +\mu_{1}\right)z-\mu_{1}+\frac{\lambda z(\mu_{1}z^{c-1}-\mu_{2})}{\mu_{2}-\mu_{1}}$ and $A_{3}\left(z\right):=\frac{\mu_{1}^{2}\left(z^{c-1}-1\right)}{\mu_{1}-\mu_{2}}$.

Using Equations (11) and (12), we obtain the PGF of the failing steady-state probabilities

$$\begin{array}{c} F\left(z\right)=\frac{\alpha_{2}}{\beta_{2}}W\left(z\right)+\left(\frac{\alpha_{1}}{\beta_{1}}-\frac{\alpha_{2}}{\beta_{2}}\right)\sum_{n=0}^{n=c-1}W_{n}z^{n}. \end{array}$$

Thus, the PGF of the system state probabilities in the steady-state

$$\begin{array}{c} P\left(z\right)=W\left(z\right)+F\left(z\right)=\left(1+\frac{\alpha_{2}}{\beta_{2}}\right)W\left(z\right)+\left(\frac{\alpha_{1}}{\beta_{1}}-\frac{\alpha_{2}}{\beta_{2}}\right)\sum_{n=0}^{n=c-1}W_{n}z^{n}. \end{array}$$

We need to determine $W_{n},n=0,1,\cdot ,c-1$, before P(z) is fully determined. Observe that z=1 is a trivial root of the denominator of W(z). Using Rouché’s theorem we can prove that the denominator has c−1 other roots inside the open unit ball (i.e., |z|<1) as shown in the following claim.

**Claim** **1.** *The denominator $z^{c}\left(\lambda +\mu_{2}\right)-\lambda z^{c+1}-\mu_{2}$ of W(z) in (25) has c−1 roots inside the open unit ball.*

**Proof.**  Define the functions $f\left(z\right):=z^{c+1}+\frac{\mu_{2}}{\lambda }$ and $g\left(z\right):=\frac{\left(\lambda +\mu_{2}\right)}{\lambda }z^{c}$. Observe that $f\left(1\right)=g\left(1\right)=1+\frac{\mu_{2}}{\lambda }$ and $f^{\prime }\left(1\right)=c+1\leq c\left(1+\frac{\mu_{2}}{\lambda }\right)=g{(}^{\prime }1)$. So, we have, for sufficiently small ϵ>0,

(26) f(1+ϵ)<g(1+ϵ).

Consider all the values of *z* on the contour |z|=1+ϵ. Using the triangle inequality and (26) we obtain

(27) $$\left|f\left(z\right)\right|\leq {\left|z\right|}^{c+1}+\frac{\mu_{2}}{\lambda }=f\left(|z|\right)=f\left(1+\epsilon \right)<g\left(1+\epsilon \right)=g\left(|z|\right)=|g\left(z\right)|,$$

and hence |f(z)|<|g(z) on the contour. Now, since both functions f(z) and g(z) are analytic on the closed disk |z|≤1+ϵ, Rouché’s theorem ensures that g(z) and g(z)−f(z) have the same number of zeros in |z|≤1+ϵ, that is, the denominator $z^{c}\left(\lambda +\mu_{2}\right)-\lambda z^{c+1}-\mu_{2}$ and $\frac{\left(\lambda +\mu_{2}\right)}{\lambda }z^{c}$ have the same number of zeros inside the closed disk |z|≤1+ϵ. Letting ϵ tend to zero yields the proof of the claim. □

These c−1 roots, ensured by the previous claim, are also the roots of the numerator due to the fact that W(z) is an analytic function on |z|≤1. Replacing these c−1 roots in the numerator, we obtain c−1 linear equations with variables $W_{i}\left(i=0,\cdots ,c-1\right)$. The *c*-th linear equation is obtained using the fact P(1)=1. This equation is equivalent to

$$a_{0}W_{0}+a_{1}W_{1}+a\sum_{n=2}^{n=c-1}W_{n}z^{n}=1,$$

where

$$\begin{array}{cc} a_{0}:=\frac{\left(\alpha_{2}+\beta_{2}\right)\left[A_{1}^{\prime }\left(1\right)+A_{2}^{\prime }\left(1\right)\right]}{\beta_{2}\left(c\mu_{2}-\lambda \right)}+\left(\frac{\alpha_{1}}{\beta_{1}}-\frac{\alpha_{2}}{\beta_{2}}\right), & a_{1}:=\frac{\left(\alpha_{2}+\beta_{2}\right)\left[A_{1}^{\prime }\left(1\right)+A_{3}^{\prime }\left(1\right)\right]}{\beta_{2}\left(c\mu_{2}-\lambda \right)}+\left(\frac{\alpha_{1}}{\beta_{1}}-\frac{\alpha_{2}}{\beta_{2}}\right),\\ a:=\frac{\left(\alpha_{2}+\beta_{2}\right)A_{1}^{\prime }\left(1\right)}{\beta_{2}\left(c\mu_{2}-\lambda \right)}+\left(\frac{\alpha_{1}}{\beta_{1}}-\frac{\alpha_{2}}{\beta_{2}}\right), \end{array}$$

with

$$A_{1}^{\prime }\left(1\right)=c\mu_{2}-\mu_{1},\;A_{2}^{\prime }\left(1\right)=\left(\lambda +\mu_{1}\right)+\frac{\lambda (c\mu_{1}-\mu_{2})}{\mu_{2}-\mu_{1}},\;A_{3}^{\prime }\left(1\right)=\frac{\left(c-1\right)\mu_{1}^{2}}{\mu_{1}-\mu_{2}}.$$

We now have *c* linear equations with *c* unknowns. The linear system of *c* equations and *c* variables is solved numerically. Once we have the values of $W_{i},i=0,\cdots ,c-1$, the value of $W_{c}$ is obtained from (7).

#### 3.1.2. Measures of Effectiveness

We now calculate some performance measures of the system using the probabilities obtained in this approach. Write $W\left(z\right)=\frac{N(z)}{D(z)}$. The expected number of customers in the system in the steady-state is

(28) $$L={\left.\frac{d}{dz}P\left(z\right)\right|}_{z=1}=\left(1+\frac{\alpha_{2}}{\beta_{2}}\right)W^{\prime }\left(1\right)+\left(\frac{\alpha_{1}}{\beta_{1}}-\frac{\alpha_{2}}{\beta_{2}}\right)S_{1}^{\prime }\left(1\right),$$

where

$$\begin{array}{ccc} W^{\prime }\left(1\right) & = & \frac{N^{\prime \prime }\left(1\right)D^{\prime }\left(1\right)-N^{\prime }\left(1\right)D^{\prime \prime }\left(1\right)}{2D^{\prime }{\left(1\right)}^{2}}, \end{array}$$

with

$$\begin{array}{ccc} D^{\prime }\left(1\right) & = & c\mu_{2}-\lambda ,\\ D^{\prime \prime }\left(1\right) & = & c\left[\left(c-1\right)\left(\mu_{2}+\lambda \right)-\lambda \left(c+1\right)\right],\\ N^{\prime }\left(1\right) & = & A_{1}^{\prime }\left(1\right)S_{1}\left(1\right)+A_{2}^{\prime }\left(1\right)W_{0}+A_{3}^{\prime }\left(1\right)W_{1},\\ N^{\prime \prime }\left(1\right) & = & A_{1}^{\prime \prime }\left(1\right)S_{1}\left(1\right)+2A_{1}^{\prime }\left(1\right)S_{1}^{\prime }\left(1\right)+\left[A_{2}^{\prime \prime }\left(1\right)+2\left(c-1\right)A_{2}^{\prime }\left(1\right)\right]W_{0}\\ & & +\left[A_{3}^{\prime \prime }\left(1\right)+2cA_{3}^{\prime }\left(1\right)\right]W_{1}, \end{array}$$

and

(29) $$A_{1}^{\prime \prime }\left(1\right)=\left(c-1\right)\left(c\mu_{2}-2\mu_{1}\right),\;A_{2}^{\prime \prime }\left(1\right)=\frac{\lambda \mu_{1}c\left(c-1\right)}{\mu_{2}-\mu_{1}},\;A_{3}^{\prime \prime }\left(1\right)=\left(c-1\right)A_{3}^{\prime }\left(1\right).$$

We may now introduce a cost function to optimize the operations of the system. Let $c_{h}$ be the unit holding cost for each customer in the system, $c_{o}$ be the operating cost per unit of time, $c_{a}$ be the startup cost per unit time for the setup of the server, and $c_{s}$ be the setup cost per busy cycle. Then, the total expected cost per unit of time is

(30) $$\begin{array}{ccc} TC(c) & = & c_{h}L+c_{o}\frac{E[B]}{E[C]}+c_{a}\frac{E[I]}{E[C]}+c_{s}\frac{1}{E[C]}, \end{array}$$

where the expected idle period, the expected busy period, and the expected busy cycle are respectively given by

(31) $$E\left[I\right]=\frac{1}{\lambda },\;E\left[B\right]=\frac{1-P_{0}}{P_{0}}E\left[I\right],\;E\left[C\right]=E\left[I\right]+E\left[B\right].$$

Thus,

(32) $$\begin{array}{ccc} TC(c) & = & c_{h}L+c_{o}+\left(\lambda c_{s}+c_{a}-c_{o}\right)P_{0}. \end{array}$$

#### 3.1.3. Illustrative Example

Take for example c=5, that is, the server adopts the single mode if four customers or less are in the system and the batch mode if five customers or more are in the system. Assume that in the single mode, the service rate is $\mu_{1}=2$, the breakdown rate is $\alpha_{1}$ = 0.05, and the repair rate is $\beta_{1}=0.07$. In the batch mode, the service rate is $\mu_{2}=5.5$, the breakdown rate is $\alpha_{2}=0.08$, and the repair rate is $\beta_{2}=0.06$. Arrivals at a rate λ=0.5. The unit costs are $c_{h}=10$, $c_{o}=20$, $c_{a}=50$, and $c_{s}=500$. The system of linear equation yields $P_{0}=0.7522$, $P_{1}=0.1875$, $P_{2}=0.0464$, $P_{3}=0.0111$, $P_{4}=0.0023$, and $P_{5}=0.0002$. Also, the average system size is L=0.3032, and the cost function has value TC=233.6479.

### 3.2. Numerical Method Using Operators

In this section, we use a different approach to find the probabilities $P_{n}$. For the sequence of probabilities $W_{n}$, we define the linear operator D by

$$W_{n}=DW_{n-1},\;\forall n\geq 1.$$

Note that composing the operators yields $W_{n+m}=D^{m}W_{n},\;\forall n,m\geq 1$.

#### 3.2.1. Computations of the Probabilities $P_{n}$

Applying this operator to Equation (8) for any 1≤n≤c−1, we get

(33) $$\zeta_{1}DW_{n-1}=\lambda W_{n-1}+\mu_{1}D^{2}W_{n-1}+\mu_{2}D^{c+1}W_{n-1}+\beta_{1}F_{n},$$

where $\zeta_{1}=\left(\lambda +\alpha_{1}+\mu_{1}\right)$. Similarly, Equation (9) gives

(34) $$\zeta_{2}DW_{n-1}=\lambda W_{n-1}+\mu_{2}D^{c+1}W_{n-1}+\beta_{2}F_{n},\;n\geq c,$$

where $\zeta_{2}=\left(\lambda +\alpha_{2}+\mu_{2}\right)$. We derive from (11) and (12)

(35) $$F_{n}=\alpha_{1}\beta_{1}^{-1}W_{n},\;1\leq n\leq c-1,$$

and

(36) $$F_{n}=\alpha_{2}\beta_{2}^{-1}W_{n},\;n\geq c.$$

Substitute (35) and (36) into (33) and (34), respectively, we obtain:

(37) $$\left[-\left(\lambda +\mu_{1}\right)D+\lambda +\mu_{1}D^{2}+\mu_{2}D^{c+1}\right]W_{n-1}=0,\;1\leq n\leq c-1,$$

and

(38) $$\left[-\left(\lambda +\mu_{2}\right)D+\lambda +\mu_{2}D^{c+1}\right]W_{n-1}=0,\;n\geq c.$$

The polynomial expressions in D in both cases give the following two characteristic equations for these difference equations:

(39) $$\mu_{2}r^{c+1}+\mu_{1}r^{2}-\left(\lambda +\mu_{1}\right)r+\lambda =0,\;1\leq n\leq c-1,$$

and

(40) $$\mu_{2}r^{c+1}-\left(\lambda +\mu_{2}\right)r+\lambda =0,\;n\geq c.$$

- Case 1: 1≤n≤c−1.

In this case we define on the interval (0,1) the function $f\left(z\right)=\mu_{2}z^{c+1}+\mu_{1}z^{2}-\left(\lambda +\mu_{1}\right)z+\lambda$. A simple study of the variations and the sign of the values of *f* gives that there exists only two real roots $r_{1}$ and $r_{2}$ of f(z)=0 on (0,1). So, for any n=1,⋯,c−1

(41) $$W_{n-1}=d_{1}r_{1}^{n-1}+d_{2}r_{2}^{n-1}\;\mathrm{and}\;F_{n-1}=\frac{\alpha_{1}}{\beta_{1}}\left(d_{1}r_{1}^{n-1}+d_{2}r_{2}^{n-1}\right),$$

where $d_{1}$ and $d_{2}$ are arbitrary constants.

- Case 2: n≥c.

In this case we define, on the interval (0,1), the function $f\left(z\right)=\mu_{2}z^{c+1}-\left(\lambda +\mu_{2}\right)z+\lambda$. Similarly to the previous case, the study of the variations and the sign of the values of *f* gives a unique real root $r_{3}$ of f(z)=0 on (0,1) which is given explicitly by $r_{3}:={\left[\frac{\lambda +\mu_{2}}{\mu_{2}\left(c+1\right)}\right]}^{\frac{1}{c}}$. So, for any n≥c

(42) $$W_{n-1}=d_{3}r_{3}^{n-1}\;\mathrm{and}\;F_{n-1}=\frac{\alpha_{2}}{\beta_{2}}\left(d_{3}r_{3}^{n-1}\right),$$

where $d_{3}$ is an arbitrary constant.

##### Calculation of $d_{1},d_{2},$ and $d_{3}$:

Given the values of the roots $r_{1},r_{2}$, and $r_{3}$ we are going to determine the values of the constants $d_{1},d_{2},$ and $d_{3}$ using the Equation (7), the Equation (9) at n=c, and the summability-to-one condition P(1)=1. We obtain the following linear system:

$$\left\{\begin{array}{ccccccc} \left(\lambda -\mu_{1}r_{1}\right)d_{1} & + & \left(\lambda -\mu_{1}r_{2}\right)d_{2} & + & \left(\mu_{2}-\mu_{1}\right)r_{3}^{c}d_{3} & = & 0\\ \left(\lambda r_{1}^{c-1}\right)d_{1} & + & \left(\lambda r_{2}^{c-1}\right)d_{2} & + & \left[\mu_{2}r_{3}^{2c}-\left(\lambda +\mu_{2}\right)r_{3}^{c}\right]d_{3} & = & 0\\ \left(\frac{1-r_{1}^{c-1}}{1-r_{1}}\right)d_{1} & + & \left(\frac{1-r_{2}^{c-1}}{1-r_{2}}\right)d_{2} & + & \left[r_{3}^{c-1}+\frac{\beta_{1}}{\beta_{1}+\alpha_{1}}\left(1+\frac{\alpha_{2}}{\beta_{2}}\right)\frac{r_{3}^{c}}{1-r_{3}}\right]d_{3} & = & \frac{\beta_{1}}{\beta_{1}+\alpha_{1}} \end{array}\right.$$

##### Calculation of $P_{n}$:

Since $P_{n}=W_{n}+F_{n}$, we readily have

$$P_{n}=\left\{\begin{array}{cc} \left(1+\frac{\alpha_{1}}{\beta_{1}}\right)\left[d_{1}r_{1}^{n}+d_{2}r_{2}^{n}\right], & 0\leq n\leq c-2,\\ \left(1+\frac{\alpha_{1}}{\beta_{1}}\right)d_{3}r_{3}^{c-1}, & n=c-1,\\ \left(1+\frac{\alpha_{2}}{\beta_{2}}\right)d_{3}r_{3}^{n}, & n\geq c. \end{array}\right.$$

Note that the numerical method gives all the probabilities $P_{n},n\geq 0$, whereas with the analytical method, we only obtain the first c+1 probabilities $P_{n},0\leq n\leq c$, and the rest of the probabilities $P_{n},n>c$, needs to be calculated by successive differentiation.

#### 3.2.2. Measures of Effectiveness

As in the other approach, we calculate the expected number of customers in the system. It is given by

$$\begin{array}{ccc} L & = & \sum_{n=0}^{\infty }nP_{n}\\ & = & \left(1+\frac{\alpha_{1}}{\beta_{1}}\right)\left[d_{1}\sum_{n=1}^{c-2}nr_{1}^{n}+d_{2}\sum_{n=1}^{c-2}nr_{2}^{n}\right]+\left(c-1\right)\left(1+\frac{\alpha_{1}}{\beta_{1}}\right)d_{3}r_{3}^{c-1}\\ & & +\left(1+\frac{\alpha_{2}}{\beta_{2}}\right)d_{3}\sum_{n=c}^{\infty }nr_{3}^{n}. \end{array}$$

The expected idle period is

(43) $$E\left[I\right]=\frac{1}{\lambda }.$$

Since we have the explicit form of $P_{0}$, we can find explicitly the mean busy period

(44) $$E\left[B\right]=\frac{\beta_{1}}{\lambda \left(\alpha_{1}+\beta_{1}\right)\left(d_{1}+d_{2}\right)}-\frac{1}{\lambda },$$

and the mean busy cycle

(45) $$E\left[C\right]=\frac{\beta_{1}}{\lambda \left(\alpha_{1}+\beta_{1}\right)\left(d_{1}+d_{2}\right)}.$$

These measures can be combined to obtain an expression for the total expected cost per unit of time

(46) $$\begin{array}{ccc} TC(c) & = & c_{h}L+c_{o}+\left(\lambda c_{s}+c_{a}-c_{o}\right)\left(d_{1}+d_{2}\right)\left(1+\frac{\alpha_{1}}{\beta_{1}}\right). \end{array}$$

#### 3.2.3. Illustrative Example

Taking the same parameter as in the previous approach, we find the probabilities $P_{0}=0.7496$, $P_{1}=0.1881$, $P_{2}=0.0472$, $P_{3}=0.0118$, $P_{4}=0.0030$, $P_{5}=0.0003$. We note that the values are remarkably close to the ones obtained in the previous example and the two methods are in total agreement. We also obtain L=0.3294 and TC=233.66.

## 4. Case Study

The queueing system studied in this paper fits perfectly the following manufacturing situation. Consider a guitar manufacturing factory where guitars can be either handmade or machine made. The two types of instruments target different market segments. Patrons select their product, basing their choice on different criteria such as quality, value and price, sound, precision, durability, long term repairability, etc. The factory operations manager has implemented a single and batch service strategy as follows: when the number of guitar orders is below some threshold level *c*, guitars are made by hand, and when it is larger than or equal to *c*, then guitars are machine made. Machine made guitars are created using a machine to replicate the look and acoustics of an authentic handmade guitar. Because there is minimal labor involved, machine made guitars can be produced quickly, and at a fraction of the price of their handcrafted originals. In this application, guitar orders are the customers. It is assumed that the time between guitar orders is exponential with parameter λ=4. The server is either the luthier or the machine. Let us assume that the service times and repair times are exponentially distributed.

A handmade guitar will carry a price which reflects its real value in terms of labor and overhead more truly than a factory made one which carries the same price. The former may take two units of time of someone’s conscientiously invested time and skill; the latter may take four to seven units of time of intensely repetitive and automated work. Assume the service rate of the luthier is $\mu_{1}^{-1}=2$ units of time and the service rate of the machine is $\mu_{2}^{-1}=5.5$ units of time. Either the luthier or the machine may be unavailable and this happens randomly with respective rates $\alpha_{1}=8$ and $\alpha_{2}=3$. Each server becomes available again after a mean time $\beta_{1}^{-1}=\frac{1}{9}$ and $\beta_{2}^{-1}=\frac{1}{6}$ units of time. The operations manager would like to know the best order level to switch from handmade to machine made guitars. The unit costs of the system are $c_{h}=10,c_{o}=20,c_{a}=50$, and $c_{s}=500$.

**Figure 2:**
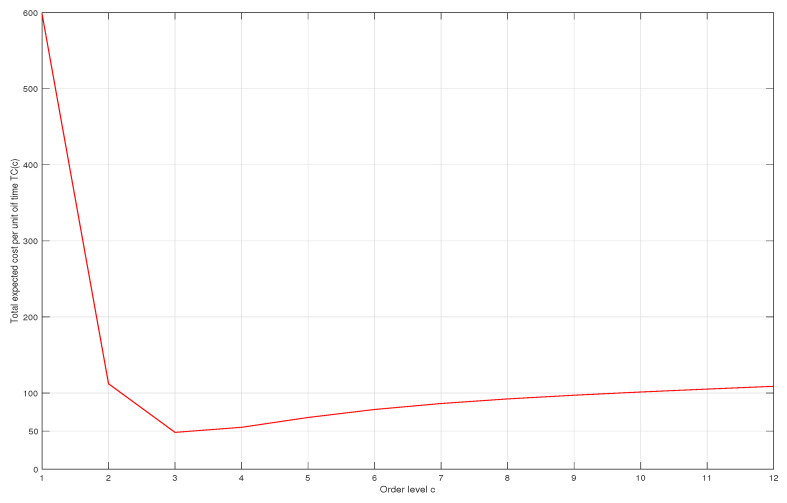
Variations of total expected cost (TC) as a function of *c*.

Applying the results obtained in the previous section, we calculate the expected total cost TC(c) for successive values of *c*, starting from c=1. The variations of the cost are represented in Figure 2. The optimal value of *c* is found to be $c^{*}=3$ and the corresponding optimal cost is $TC^{*}=48.3038$. In managerial terms for the operations manager, this means that the optimal policy is to have the luthier make the guitars by hand as long as there are less than three orders in line. If this number is grater than or equal to 3, then guitars should be made using the machine.

In case there is some uncertainty about some of the parameters, a sensitivity analysis can be conducted to assess the effect of this uncertainty on the optimal policy. For example, suppose there is an uncertainty about $\alpha_{1}$, the failure rate when the server is in the working state. Then optimal measures can be calculated for various values of $\alpha_{1}$. A sample calculations is shown in Table 1 where we calculate the probability of no orders $P_{0}$, the average number of orders *L*, and the optimal expected cost per unit of time $TC^{*}$.

The effect of other parameters can be assessed in a similar way.

## 5. Conclusions

The Markovian queueing system considered in this paper is characterized by a flexible server that adapts to the queue length by switching from a single service to a bulk service when the queue length is too large and from bulk service to single service when the queue length is too small. The server is unreliable and may break down while providing service. Different parameters depend on the service discipline applied. We calculated the system size steady-state probabilities in terms of their probability generating function and using linear operators. The two methods comply with each other. An application to a case study is also provided.

There are various ways this work can be further developed. For example, bulk arrivals instead of single arrivals could be examined. Also general distributions could be assumed for the various processes considered. Server vacations, either working or not, and various threshold policies such as N-, T-, or D-policies could also be taken into account.

## Figures and Tables

**Table 1:** Sensitivity to the breakdown rate $\alpha_{1}$.

$\alpha_{1}$	1	2	3	4	5	6	7	8	9	10
$P_{0}$	0.1646	0.1671	0.1693	0.1712	0.1728	0.1743	0.1756	0.1767	0.1778	0.1787
L	2.3822	2.3435	2.3103	2.2815	2.2563	2.2341	2.2143	2.1967	2.1807	2.1664
$TC^{*}$	377.92	382.69	386.78	390.32	393.42	396.15	398.58	400.76	402.71	404.48
